# A longitudinal study of fatty acid profiles, macronutrient levels, and plasmin activity in human milk

**DOI:** 10.3389/fnut.2023.1172613

**Published:** 2023-05-09

**Authors:** Fanyu Meng, Therese Uniacke-Lowe, Elisa Lanfranchi, Grainne Meehan, Carol-Anne O'Shea, Theresa Dennehy, Anthony C. Ryan, Catherine Stanton, Alan L. Kelly

**Affiliations:** ^1^School of Food and Nutritional Sciences, University College Cork, Cork, Ireland; ^2^Department of Neonatology, Cork University Maternity Hospital, Cork, Ireland; ^3^Brookfield School of Medicine and Health, University College Cork, Cork, Ireland; ^4^Teagasc Food Research Centre, Moorepark, Cork, Ireland; ^5^APC Microbiome, Cork, Ireland

**Keywords:** human milk, infant nutrition, macronutrients, plasmin, fatty acids

## Abstract

**Introduction:**

Human milk provides nutrients essential for infant growth and health, levels of which are dynamic during lactation.

**Methods:**

In this study, changes in macronutrients, fatty acids, and plasmin activities over the first six months of lactation in term milk were studied.

**Results:**

There was a significant influence of lactation stage on levels of protein and plasmin activities, but not on levels of fat and carbohydrate in term milk. Concerning fatty acids in term milk, levels of caproic acid and α-linolenic acid increased significantly (*p* < 0.05), whereas those of arachidonic acid and docosahexaenoic acid decreased, in the six months after birth. Significant impacts of maternal pre-pregnancy BMI and infant gender on fatty acid profiles were also found. Multivariate statistical analysis showed that protein level, plasmin activity, and several fatty acids (α-linolenic acid, lignoceric acid, and docasadienoic acid) contributed strongly to discrimination of milk from different lactational stages.

**Discussion:**

The study demonstrates that not all but some fatty acids were influenced by lactation, whereas protein and protease levels showed clear decreasing trends during lactation, which may help in understanding the nutritional requirements of infants.

## Introduction

Human milk contains essential macro- and micro-nutrients and is rich in bioactive components, which provide short-term and long-term benefits to infants (1). Macronutrients in human milk, including protein, fat, and carbohydrate are essential to infant growth and development. Besides macronutrients, human milk also provides enzymes that break down macronutrients to aid infant digestion. Proteases that hydrolyse the peptide bonds in proteins and peptides present in human milk include plasmin, trypsin, kallikrein, cathepsins, elastase, thrombin, amino- and carboxypeptidases and matrix metalloproteinases (2, 3). Among these, plasmin has been reported to be active at the pH of the infant stomach and is the major protease that hydrolyses caseins, as well as some proteins in whey, such as polymeric immunoglobulin receptor and osteopontin (4). Therefore, plasmin may potentially influence infant growth in both nutritional and immune functions. Studies on changes in plasmin activity during lactation can help understanding the dynamics of human milk and the requirement for infants; however, knowledge in this area is still limited (5, 6).

Moreover, fatty acids (FAs) in human milk are of significance because they contribute to 95% (w/w) of neutral lipids (7). The composition of FAs can largely influence the properties of milk triglycerides, thereby influencing the energy intake and infant growth (8). Meanwhile, FAs play roles as both the precursors of important metabolic compounds and the building blocks of cell membrane (9). Human milk is also a rich source of n-3- and n-6-polyunsaturated fatty acids (n-6 PUFA and n-3 PUFA) and their long-chain PUFA (LC PUFA) derivatives, such as arachidonic acid (AA) and docosahexaenoic acid (DHA), which have been demonstrated to promote neonatal growth and brain development (10).

The levels of nutrients in human milk are dynamic and have been reported to be influenced by many factors, including lactational stages, gestational age, maternal diet, age of mothers, parity, maternal body mass index (BMI), infant birth weight, infant gender, and diurnal variation (11–13). However, the impact of those factors on human milk composition is still controversial. For example, concerning the lactational duration, the level of fat has been reported to increase in early lactation (14, 15) and then remain stable (16, 17). However, one recent study from Netherlands reported a decrease in fat and a significant decrease in energy from 1 to 3 months (18). In the same way, the levels of carbohydrate have been reported to increase during the early stages of lactation (~1-month post-partum) and remain constant afterwards (16, 19), remain stable from the first week (5, 17) or increase from 1 to 6 months (20) accroding to studies from Western countries.

Studies have also demonstrated a sex-bias effect on human milk composition, but this is still under researched (21, 22). Some studies reported higher levels of carbohydrate (15), fat, and energy in milk for male infants (17, 23), while others reported increased carbohydrate and energy levels in milk for female infants (24). In contrast, no significant effect of infant gender on macronutrients in milk has been reported (25). Some studies have also reported that the gender of a baby affects other components in human milk, including minerals and hormones, and the milk microbiome (21, 26, 27).

Differing results of the impact of maternal BMI on macronutrients have also been reported; some studies have reported that lactose levels were lower (28, 29) and fat level was higher (30–32) in milk from mothers with BMI ≥25 kg m^−2^, while others found no association between BMI and macronutrient levels in human milk (29, 33). Moreover, the influencing factors on FA composition, such as maternal BMI and infant gender remain unclear. Therefore, this study attempts to provide more evidence on how such factors influence the FAs.

This study reports the changes in milk composition, including macronutrients, plasmin activities, and FA profiles up to 6 months of lactation; the influence of factors including maternal BMI and infant gender on milk macronutrients and FA profiles are also examined.

## Methods

### Ethical approval and study design

This study was approved by the Clinical Research Ethics Committee of the Cork Teaching Hospitals. All participants were recruited at Cork University Maternity Hospital and provided written consent during the defined period of time of the study (Jan 2017 to Dec 2020), and all relevant guidelines and regulations were followed.

**Figure 1:**
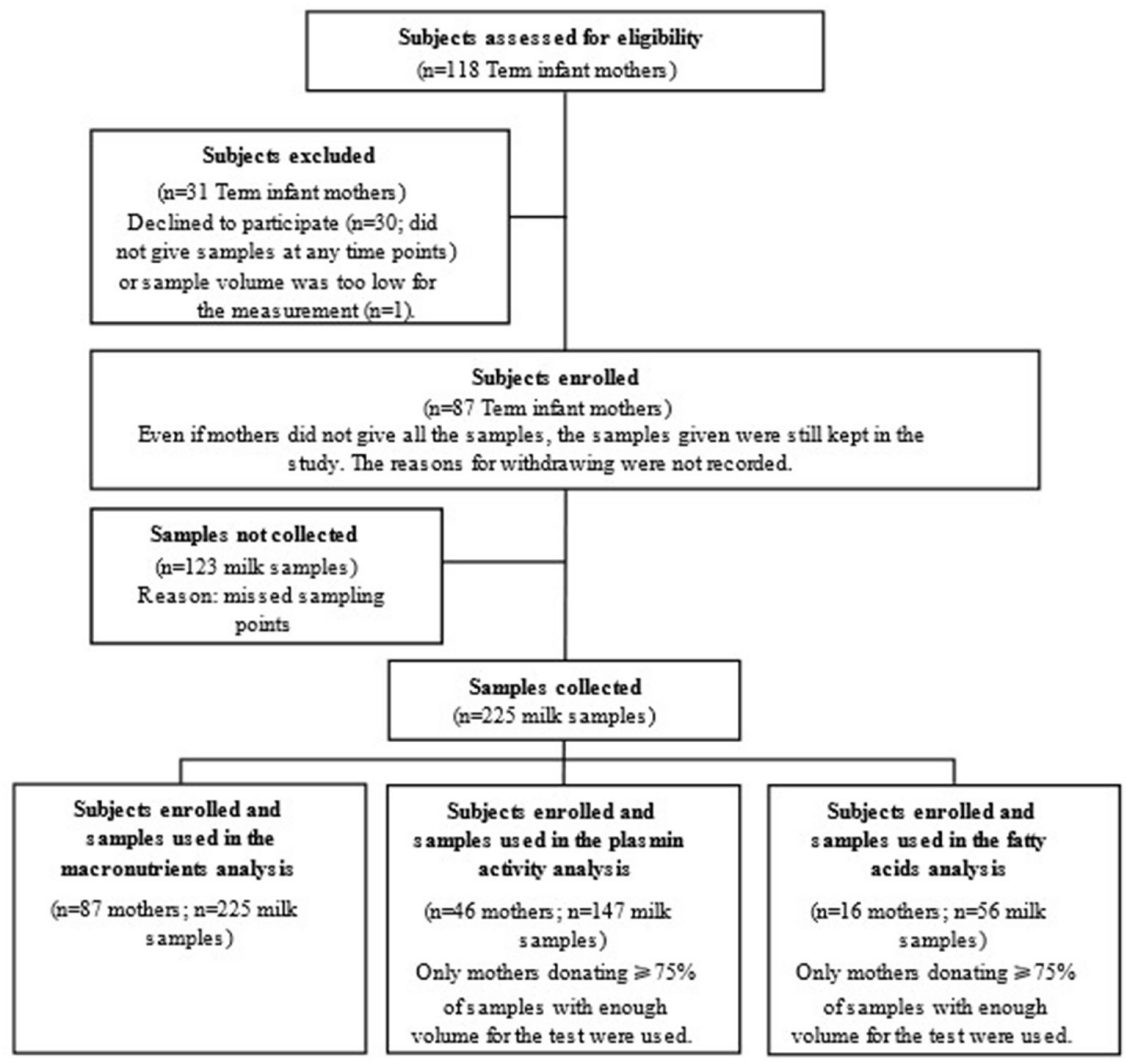
Study flow chart indicating subject recruitment and sample collection for analyses in the study.

Healthy mothers who had given birth at full term delivery (118 recruited, 31 excluded) were enrolled in this study (Figure 1). The sample size was similar to previous studies and the number of subjects was sufficient for our study (19, 24). Term milk was collected at 1-, 4-, 8-, and 24-weeks *post-partum*. Questionnaires on 24-h dietary recall were collected with the milk samples.

### Human milk collection and storage

For milk sampling, mothers expressed 10–20 ml of milk, which was collected after the infant was satisfied, and hence represented largely mid- to hind-milk. Milk was collected into a sterile container provided and stored at 4°C until delivery to the laboratory. Samples for macronutrient, plasmin activity and FA analysis were stored at −80°C until analysis. All chemicals were obtained from Merck KGaA, Darmstadt, Germany, unless otherwise stated.

### Determination of levels of macronutrients

Total fat, protein, carbohydrate, and energy contents of human milk were determined with a human milk analyser (MIRIS, Uppsala, Sweden) based on mid-infrared transmission spectroscopy. The levels of energy were calculated by MIRIS as: Energy (kcal L^−1^) = protein (g L^−1^) × 4.4 + fat (g L^−1^) × 9.25 + carbohydrate (g L^−1^) × 4. Samples collected for compositional analysis were thawed at 4°C overnight, warmed to 40°C and homogenized using a MIRIS sonicator (MIRIS, Uppsala, Sweden) before analysis. Approximately 1.5 ml of human milk was injected into the human milk analyser and the measurement of each sample was in duplicate.

### Determination of plasmin activity

Plasmin activity in milk was measured in duplicate using a modified version of the method of Richardson and Pearce (34). Human milk samples were thawed at 4°C overnight and centrifuged at 14,000 rpm for 20 min with 0.4 M trisodium citrate added (3:1 ratio) to obtain a clear serum phase. A reaction mix of the sample (100 μl), coumarin substrate (40 μl) and 50 mM tris-HCl buffer (pH 7.5, 60 μl) were added into a well of black 96-well microplates (VWR International Ltd, Luttervorth, UK). The fluorescence intensity was determined at 380 nm (excitation) and 460 nm (emission) using a plate reader (Spectrafluorplus, Tecan). One unit of plasmin activity is defined as the activity necessary to release 1 nmol 7-amino-4-methyl coumarin (AMC) per ml per min under the conditions of the assay.

### Determination of fatty acids in human milk

Prior to the analysis, milk samples were thawed at 4°C overnight and inverted 20 times to achieve a good mixing of milk fats. Total FAs were extracted from human milk and fatty acid methyl esters (FAME) were prepared according to the method of Kelishadi et al. (35) with some modifications. Briefly, 1 ml of internal standard (1 mg ml^−1^ of C11:0 in methanol) was added to 1 ml milk followed by 0.7 ml of 10 N KOH and 5.3 ml of methanol in a screw-threaded clear glass tube. The tube was incubated in a water bath at 55°C for 1.5 h and vortexed for 5 s at 20 min intervals. Samples were cooled to below room temperature with ice water and 0.58 ml of 24 N H_2_SO_4_ was added. Tubes were mixed by inversion and incubated at 55°C as before for 1.5 h, vortexing for 5 s every 20 min. Tubes were then cooled and 2 ml of hexane was added. Each sample was vortexed for 2 min followed by centrifugation (Beckman J-E Highspeed, Beckman Coulter, Brea, California) for 2 min at 5,000 rpm at 4°C. The clear upper phase (FAME extract) was transferred to a screw-cap Pyrex tube with Na_2_SO_4_ (1 g) added to remove water, followed by transfer to the GC test vial. Vials were capped and stored at −80°C until gas chromatography (GC) analysis.

A Supelco 37 Component FAME mix (product CRM47885) was used as stock solution to identify and quantify FAs in milk samples. Calibration curves were produced for each individual fatty acid from 5 working standards prepared by diluting the stock solution with hexane (10, 7, 5, 2, and 1 mg ml^−1^ standard mixes). Calibration curves consisted of a plot of peak area vs. concentration. The response linearity of each fatty acid had *r*^2^ values higher than 0.99. The calibration was validated and tested between batches of 20 samples. The limit of quantitation and limit of detection were established using the lowest concentration standard, where all FAME compounds assessed exhibited signal-to-noise (S/N) values of at least 11. Undecanoic acid (C11:0) was used as internal standard to monitor the efficiency of the FA extraction process and to calculate recovery rates. The latter was performed by analyzing and comparing the 5 mg ml^−1^ working standard [1] spiked with C11:0, at a concentration similar to that added to samples [2] and C11:0 analyzed alone at this concentration [3]. In all cases, the area counts from [2] and [3] were similar at > 95%.

GC analysis of FAMEs was carried out with a Shimadzu GC-2010 Plus Gas Chromatograph (Shimadzu Scientific Instruments, Kyoto, Japan), equipped with a split/splitless injector, an AOC-20i autoinjector, an AOC-20s autosampler, a flame ionization detector (FID) and a high polarity cyanopropyl siloxane phase-based column (Agilent J&W CP-Sil 88 for FAME), 50 m × 0.25 mm with 0.2-μm film thickness (Agilent Scientific Instruments, Santa Clara, California). Helium was used as carrier gas at a linear velocity of 0.5 ml min^−1^. Air and hydrogen flow rates were set at 400 and 40 ml min^−1^, respectively. The make-up flow rate was set at 30 ml min^−1^, and the auto injector and the detector temperatures were set at 260 and 270°C, respectively. Two microliter of sample was injected, with the split set at 1/100. The column oven temperature was maintained at 30°C for 2 min, then increased at 10°C min^−1^ to 175°C and held for 15 min, followed by increasing at 5°C min^−1^ to 205°C, and finally by increasing at 3°C min^−1^ to 220°C and holding at this temperature for 24.5 min. The total runtime was 75 min. LabSolutions software (Shimadzu Scientific Instruments) was used for peak area analysis and quantification of FAs. Samples were analyzed in duplicate and the results for each FA were expressed as percentage of total FAs/peak area (%). The results for summed concentrations of saturated fatty acids (SFA), monounsaturated fatty acids (MUFA), n-3 LC PUFA, and n-6 LC PUFA are expressed as mg g^−1^ of total fat in a milk sample. A hexane blank was run between every 4 samples to ensure no carryover between samples.

### Statistical analysis

Univariate statistical analyses of results were conducted with SPSS software version 17.0 (IBM, Armonk, New York). A mixed linear model was applied with the lactational stage (as categories) to determine whether the macronutrients, plasmin activity, and FAs changes over lactation time and to assess the impact of following factors on human milk composition: maternal pre-pregnancy BMI and infant gender. To estimate the effect of infant gender during lactation, the lactational stage (as categories), infant gender and their interaction were considered as fixed effects and the subject identification was considered as a random effect. A similar method was used to estimate pre-pregnancy BMI (as categories: BMI < 25 kg m^−2^ and BMI ≥ 25 kg m^−2^) and the interaction with age. Bonferroni *post hoc* tests with a level of significance at *p* < 0.05 were applied to analyse difference between groups. The data for each nutrient was tested for normality according to *Q*–*Q* plots and Kolmogorov–Smirnova test. Logarithmic transformation was applied to each nutrient when the data were not normally distributed.

To reduce the dimensionality of the multivariate dataset and to discriminate difference between samples from different lactational stages, principal component analysis (PCA) and partial least squares—discriminant analysis (PLS-DA), were also employed using SIMCA software version 14.1.0 (Umetrics AB, Umeå, Sweden). Unit variance auto scaling was used on the data obtained from deconvolution methods. PCA used orthogonal transformation to convert a set of potentially correlated variables into a set of values of linearly uncorrelated variables (principal components; PCs) that explain a large portion of the variance in the dataset. The PLS-DA model was computed to identify associations between the data (*X* variable) and lactational groups (*Y* variable). The model was validated using *k*-fold cross validation (*k* = 7) and a permutation test (200 permutations) (36), which measured its performance based on its *R*^2^ and *Q*^2^ values, as well as its accuracy.

## Results

### Participants

In total, 87 mothers who gave birth to full term neonates were enrolled in this study. As shown in Table 1, the average gestational age (mean ± SD) at birth was 39.5 ± 1.0 weeks, and the average mothers' pre-pregnancy BMI was close to the healthy weight threshold, i.e., 25 kg m^−2^. Meanwhile, the ratios of female infants to male infants was nearly 1:1 for the full study population, as well as for the sub-groups of participants, i.e., participants involved in the assay of FA profiles and the plasmin activity.

**Table 1:** Key demographic and infant data for participants in the study.

**Variables**	**Parameters**		
	**Macronutrients (*****N*** = **87)**	**Plasmin activity (*****N*** = **46)**	**Fatty acids profile (*****N*** = **16)**
Infant gestational age (week)	39.5 (1.0)	39.6 (0.9)	39.3 (0.8)
Infant birth weight (g)	3,635.1 (436.5)	3,625.8 (386.8)	3,469.4 (274.3)
Infant gender (% female)	47.1	52.2	50.0
Mothers' BMI pre-pregnancy (kg/m^2^)	26.5 (8.1)^*^	25.6 (5.2)	25.5 (4.5)
Mothers' BMI pre-pregnancy (% BMI < 25 kg m^−2^)	53.3	55.6	66.7
Mode of delivery (% C-section)	21.8	23.9	25.0

^*^Results are presented as average (standard deviation).

The total number of milk samples collected decreased during lactation as the number of mothers who stopped breast-feeding increased (Supplementary Table S1). In mothers who continued breast-feeding to 6 months, exclusive breast-feeding decreased from 82 to 56% during 24 weeks of lactation, as frequently seen (37).

### Macronutrient levels during lactation

**Figure 2:**
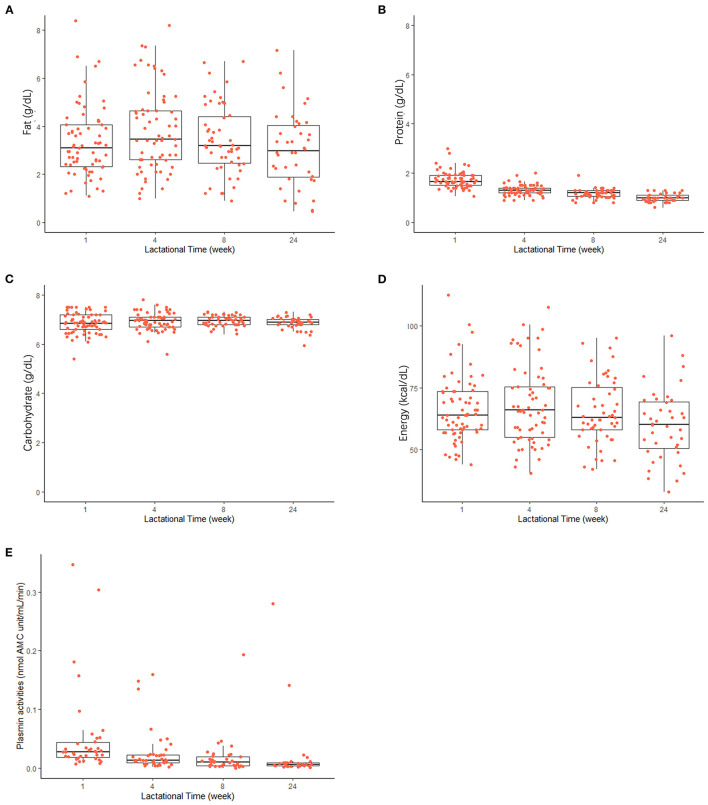
Changes in macronutrient levels [**(A)** fat; **(B)** protein; **(C)** carbohydrate], energy content **(D)**, and plasmin activities **(E)** in human milk during the first 24 weeks of the lactation. Horizontal lines inside boxes indicate the median value. Lines extending vertically from the boxes indicate variability outside the upper and lower quartiles are indicated. Red points indicate the individual values in each lactational time group.

As shown in Figure 2, levels of protein (Mean ± SD) in term milk decreased significantly (*p* < 0.05, *n* = 87), from 1.7 ± 0.3 to 1.0 ± 0.2 g dl^−1^ from the first week to the 6 months after birth. The average total fat in term milk at each lactational stage did not show clear trends (ranged from 3.0 ± 1.8 g dl^−1^ at 24 weeks to 3.8 ±1.7 g dl^−1^ at 4 weeks). Carbohydrate content remained relatively unchanged during lactation (6.8–7.0 g dl^−1^). Energy content showed a similar trend to the fat content during lactation, in that the highest energy content was at 4 weeks *post-partum*; however, there was a significant association with lactation stage (*p* < 0.05, *n* = 87).

In addition, the impact of maternal BMI and infant gender on macronutrient levels in milk was estimated (Supplementary Figures S1, S2). Maternal BMI significantly (*p* < 0.05, *n* = 75) influenced levels of protein in milk at 4 weeks after birth; the protein level was lower in milk expressed by mothers with low BMI than the milk expressed form high BMI mothers after 4 weeks *post-partum*. No gender effect was found on levels of macronutrients and energy in milk at each lactational time point (*p* > 0.05, *n* = 87).

### Plasmin activities

As shown in Figure 2E, plasmin activity decreased during lactation in human milk, with high inter-individual differences, especially at early stages after birth. Plasmin activity significantly decreased from 0.052 ± 0.07 nmol AMC units min^−1^ ml^−1^ to 0.026 ± 0.04 nmol AMC units min^−1^ ml^−1^ at 4 weeks *post-partum* (*p* < 0.05, *n* = 46) and remained relatively constant after that.

### Fatty acid profiles

**Figure 3:**
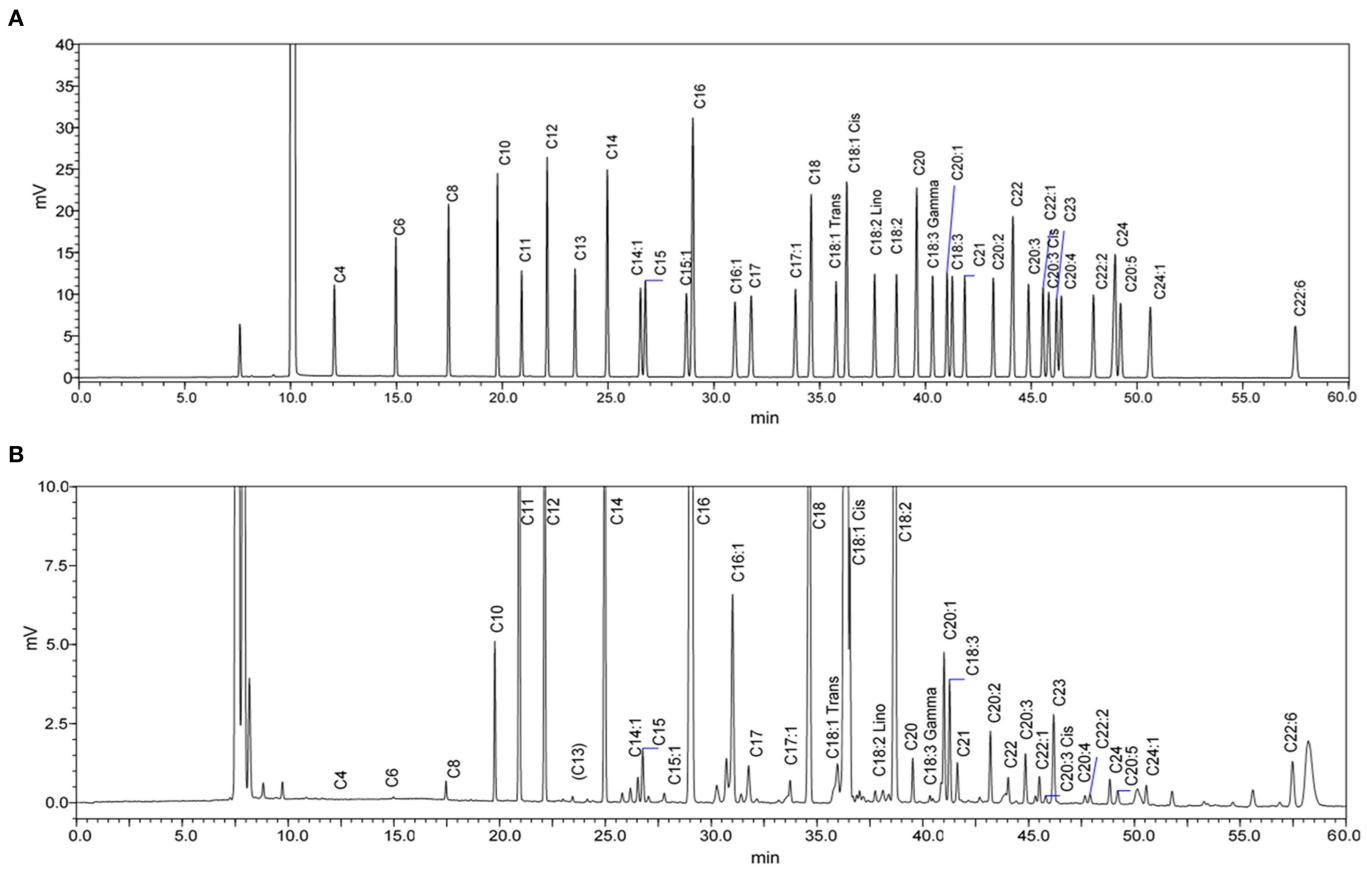
GC-FID chromatograms of **(A)** Supelco 37-Component fatty acid methyl esters (FAME) standard mix and **(B)** FAME from a human milk sample after one-week *post-partum*. The chromatogram of the milk sample is scaled to show all significant peaks.

GC chromatograms of the standard mix and an example of human milk FAs are shown in Figure 3; separation was achieved with good resolution. Among 35 FAs measured, summed concentrations of SFA, MUFA, n-3 LC PUFA, and n-6 LC PUFA are summarized in Table 2; these were not significantly influenced by lactation (*p* > 0.05, *n* = 16), i.e., they remained relatively constant during lactation. ∑ SFA (mean ± SD) content was 160.4 ± 42 to 164 ± 23 mg g^−1^ total fat, which was higher than ∑ MUFA, which was measured as 104.4 ± 52 to 117.6 ± 43 mg g^−1^ total fat. In terms of LC PUFAs, levels of ∑ n-3 LCPUFAs were lower than ∑ n-6 LCPUFAs.

**Table 2:** Concentrations (mg g^−1^ total fat) of the sum of saturated fatty acids, monounsaturated fatty acids, n-3 long-chain poly-unsaturated fatty acids, and n-6 long-chain polyunsaturated fatty acids in human milk during lactation.

**Lactational time**	**Concentrations (mg g**^**−1**^ **total fat)**			
	**Week 1 (*****N*** = **16)**	**Week 4 (*****N*** = **14)**	**Week 8 (*****N*** = **10)**	**Week 24 (*****N*** = **16)**
SUM SFA	162.1 (35.7)^*^	162.6 (23.6)	160.4 (41.7)	164.0 (28.3)
SUM MUFA	117.6 (43.8)	113.6 (32.5)	109.7 (49.5)	104.4 (52.3)
SUM n-3 LCPUFAs	7.3 (1.7)	7.5 (1.1)	7.4 (2.3)	7.5 (1.7)
SUM n-6 LCPUFAs	36.9 (15.9)	36.0 (6.1)	37.4 (11.7)	38.1 (9.1)
Others (not defined)	676.2 (80.4)	680.3 (41.2)	685.1(67.2)	686.0 (57.8)

SFA, saturated fatty acids; MUFAs, monounsaturated fatty acids; LCPUFAs, long-chain polyunsaturated fatty acids.

^*^Results are presented as average (standard deviation).

Average concentrations of individual FA in milk are shown in Table 3. According to the percentage of total FAs/peak area in GC chromatograms (see the example in Figure 3B), the most abundant FA (% total FAs) in term human milk out of 35 FAs measured in this study was oleic acid (C18:1 n-9; 28–31%). Other abundant FAs (% total FAs) in term milk included palmitic acid (C16:0; 20–21%), linoleic acid (C18:2 n-6; 10–11%), stearic acid (C18:0; 6.4–6.9%), myristic acid (C14:0; 5.8–6.4%), and lauric acid (C12:0; 5–5.1%).

**Table 3:** Fatty acid profiles of human milk during lactation.

**Lactational time**	**% Total fatty acids/peak area**			
	**Week 1 (*****N*** = **16)**	**Week 4 (*****N*** = **14)**	**Week 8 (*****N*** = **10)**	**Week 24 (*****N*** = **16)**
**Saturated fatty acids (SFAs)**				
C6:0	0.00 (0.0)^a*^	0.00 (0.0)^ab^	0.01 (0.0)^ab^	0.02 (0.0)^b^
C8:0	0.10 (0.0)	0.14 (0.0)	0.14 (0.0)	0.11 (0.0)
C10:0	0.95 (0.3)	1.22 (0.3)	1.17 (0.3)	1.00 (0.2)
C12:0	4.89 (1.7)	5.08 (1.5)	5.05 (1.6)	5.11 (1.5)
C13:0	0.03 (0.0)	0.03 (0.0)	0.03 (0.0)	0.02 (0.0)
C14:0	5.79 (1.7)	5.79 (1.1)	5.80 (1.0)	6.39 (1.7)
C15:0	0.30 (0.1)	0.32 (0.1)	0.34 (0.1)	0.29 (0.1)
C16:0	21.08 (2.6)	20.84 (2.4)	20.38 (2.9)	19.68 (2.5)
C17:0	0.33 (0.1)	0.32 (0.1)	0.33 (0.1)	0.30 (0.1)
C18:0	6.38 (1.4)	6.77 (1.4)	6.88 (2.2)	6.66 (0.8)
C20:0	0.20 (0.0)	0.20 (0.0)	0.19 (0.1)	0.17 (0.0)
C21:0	0.25 (0.1)	0.30 (0.1)	0.31 (0.1)	0.32 (0.1)
C22:0	0.16 (0.2)	0.24 (0.2)	0.33 (0.2)	0.14 (0.1)
C23:0	0.57 (0.1)^a^	0.42 (0.1)^b^	0.43 (0.1)^b^	0.39 (0.1)^b^
C24:0	0.08 (0.0)^a^	0.04 (0.0)^ab^	0.03 (0.0)^ab^	0.02 (0.0)^b^
**Monounsaturated fatty acids (MUFAs)**				
C14:1	0.17 (0.1)	0.21 (0.1)	0.23 (0.1)	0.18 (0.1)
C15:1	0.05 (0.0)	0.06 (0.0)	0.06 (0.0)	0.05 (0.0)
C16:1	1.90 (0.6)	2.02 (0.6)	2.31 (0.8)	2.13 (0.6)
C17:1	0.18 (0.0)	0.17 (0.0)	0.18 (0.0)	0.16 (0.1)
C18:1 n-9	31.35 (11.5)	31.09 (9.5)	30.12 (13.4)	28.10 (15.7)
trans-C18:1 n-9	0.56 (0.4)	0.62 (0.3)	0.52 (0.4)	0.55 (0.5)
C20:1	0.62 (0.1)^a^	0.47 (0.1)^b^	0.46 (0.1)	0.44 (0.1)^b^
C22:1	0.13 (0.0)^a^	0.08 (0.0)^ab^	0.07 (0.0)^b^	0.07 (0.0)^b^
C24:1	0.19 (0.1)	0.25 (0.2)	0.25 (0.2)	0.33 (0.2)
**n-3 long-chain polyunsaturated fatty acids (n-3 LCPUFAs)**				
C18:3 n-3	1.01 (0.3)^a^	1.27 (0.3)^ab^	1.28 (0.4)^ab^	1.36 (0.3)^b^
C20:3 n-3	0.08 (0.0)^b^	0.04 (0.0)^ab^	0.04 (0.0)^ab^	0.03 (0.0)^b^
C20:5 n-3	0.06 (0.0)	0.08 (0.0)	0.08 (0.0)	0.05 (0.0)
C22:6 n-3	0.41 (0.1)^a^	0.28 (0.1)^ab^	0.29 (0.1)^ab^	0.21 (0.1)^b^
**n-6 long-chain polyunsaturated fatty acids (n-6 LCPUFAs)**				
C18:2 n-6	9.94 (2.4)	10.32 (2.3)	11.03 (3.4)	11.07 (2.7)
trans-C18:2 n-6	0.08 (0.0)	0.07 (0.0)	0.08 (0.0)	0.07 (0.0)
C18:3 n-6	0.06 (0.0)	0.11 (0.0)	0.10 (0.0)	0.08 (0.0)
C20:3 n-6	0.41 (0.1)	0.40 (0.1)	0.34 (0.1)	0.26 (0.1)
C20:4 n-6	0.10 (0.1)^a^	0.12 (0.0)^a^	0.10 (0.0)^a^	0.06 (0.0)^b^
**Other polyunsaturated fatty acids (PUFAs)**				
C20:2	0.42 (0.1)^a^	0.26 (0.1)^ab^	0.22 (0.1)^b^	0.18 (0.1)^b^
C22:2	0.07 (0.0)^ab^	0.03 (0.0)^b^	0.03 (0.0)^b^	0.02 (0.0)^b^

Significant differences between time points are indicated by different letters.

^*^Results are presented as average (standard deviation).

There was a significant increase (*p* < 0.05) in levels of caproic acid (C6:0) and α-linolenic acid (ALA; C18:3 n-3) during lactation (Table 3). In contrast, levels of long-chain FAs, including AA (C20:4 n-6), erucic acid (C22:1), docasadienoic acid (C22:2), DHA (C22:6), and lignoceric acid (C24:0) decreased significantly (*p* < 0.05) during lactation. In addition, the ratio of linoleic acid to AA decreased from 9.8 to 8.1 during the first 6 months of lactation. Concentrations of cis-11-eicosenic acid (C20:1) and tricosanoic acid (C23:0) decreased significantly (*p* < 0.05) during the first 4 weeks after birth, but thereafter remained consistent until 24 weeks after birth. Lactational stage also had a significant impact on γ-linolenic acid (C18:3 n-6) levels (*p* < 0.05), the concentrations of which increased during 1–4 weeks after birth, but decreased from 4 to 24 weeks after birth; a summarized table of significantly changed fatty acids can be seen in Table 4.

**Table 4:** A summary of the fatty acids with significantly changed fatty acids during the first 6 months of lactation.

**Factors**	**Fatty acids**	**Our study**	**Other studies**	**References**
Lactation	C6:0	↑	↑	(38)
	C18:3 n-3	↑	↑	(38)
	C20:4 n-6 (AA)	↓	↓	(38–40)
	C22:1 n-9	↓	↓	
	C22:2	↓	↓	
	C22:6 (DHA)	↓	↓	
	C24:0	↓	↓	
	***Cis*** **-C20:1** ^a^	↓First 4 weeks after birth	–	–
	**C23:0**	↓First 4 weeks after birth	–	–
	**C18:3 n-6**	↑First 4 weeks after birth; ↓4 weeks to 24 weeks after birth	–	–
BMI	C8:0	Higher in high BMI group^b^ (8–24 weeks after birth)	Higher in BMI ≥30 kg m^−2^ group	(41)
	C15:0	Higher in high BMI group (4–8 weeks after birth)	Higher in BMI ≥30 kg m^−2^ group	
	**C14:1**	Higher in high BMI group (4–24 weeks after birth)	–	–
	**C24:1**	Higher in high BMI group (8–24 weeks after birth)	–	–
	**C22:2**	Lower in high BMI group (4–8 weeks after birth)	–	–
Gender	**C8:0**	Higher in female infant group (24 weeks after birth)	–	–
	**C14:1**		–	–
	***trans*** **-C18:1 n-9**		–	–
	**C24:1**		–	–
	C18:3 n-3	Higher in female infant group (8 weeks after birth)	Higher in female infants group	(42)

^a^Fatty acids with bold fonts: trends have not been reported in previous references.

^b^High BMI group: milk from mothers with BMI ≥ 25 kg m^−2^.

↑: fatty acids increased with lactation; ↓: fatty acids decreased with lactation.

The bold text indicates that no reports of the same trend from other studies.

In addition, maternal BMI had a significant impact (*p* < 0.05) on six SFAs (C8:0, C14:0, C15:0, C17:0, C21:0, and C24:0), five MUFAs (C14:1, C15:1 C16:1 C17:1, and C24:1), and six PUFAs (trans C18:2 n-6, C18:3 n-6, C20:3 n-6, C20:4 n-6, C20:5 n-3, and C22:2) in term milk (Supplementary Figure S3). Significantly (*p* < 0.05, *n* = 16) higher levels of most FAs were present in high BMI mothers (BMI ≥ 25 kg m^−2^) at week 4, week 8, or week 24. In contrast, term milk from high-BMI mothers contained significant (*p* < 0.05) lower levels of lignoceric acid (C24:0) at week 8, and lower levels AA (C20:4 n-6) and EPA (C20:5 n-3) at week 24.

When considering the impact of gender at each lactation stage (Supplementary Figure S4), there is no significant difference in each FA between milk of mothers of male or female infants after 1 or 4 weeks after birth. Significantly higher levels (*p* < 0.05, *n* = 16) of ALA (C18:3 n-3) and ∑ n-3 LCPUFAs were present in milk for female infants than in milk for male infants after 8 weeks *post-partum*. In milk collected 24 weeks *post-partum*, concentrations of SFAs, including caprylic acid (C8:0), myristic acid (C14:0), and ∑ SFA, as well as some MUFAs, including myristoleic acid (C14:1), nervonic acid (C24:1), and trans-9-elaidic acid (trans C18:1 n-9), were significantly higher in milk from mothers of female infants than in milk for male infants.

### Multivariate statistical analysis

**Figure 4:**
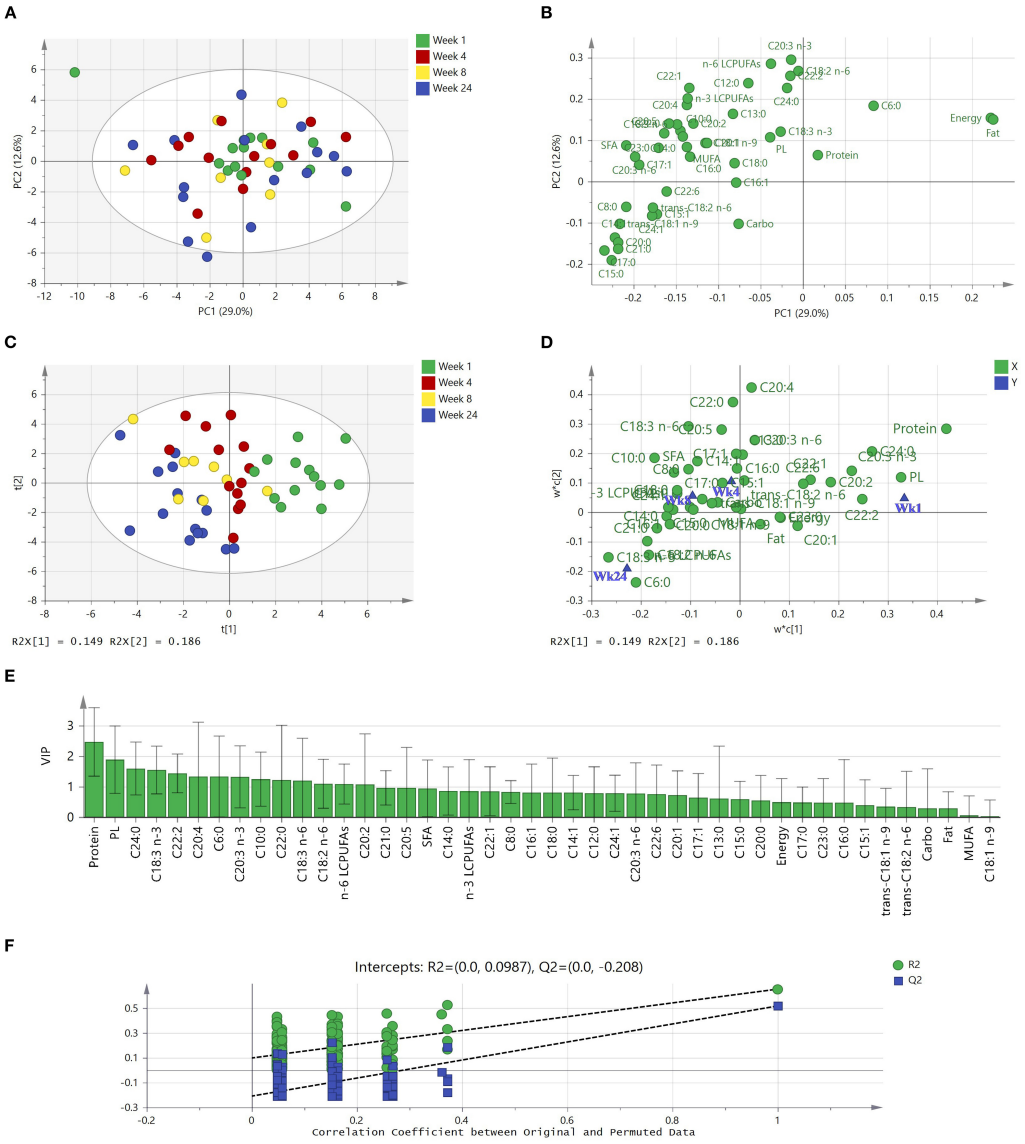
PCA and PLS-DA of subgroup human milk samples (*n* = 50) from term deliveries collected during the first 24 weeks of lactation. **(A)** PCA scores scatter plot of observations colored according to lactational stages. **(B)** Corresponding PCA loadings scatter plot of variables measured. **(C)** PLS-DA scores scatter plot of observations collected colored according to lactational stages (one extreme outlier was excluded). **(D)** Corresponding w*c loadings scatter plot applies both the *X*-weights (w*) and *Y*-weights (c) to identify associations between the collected data (*X* variables) and lactational stages (*Y* variables). **(E)** *X* variables (macronutrients, plasmin activities, and fatty acids) able to discriminate between lactational groups, ordered by VIP score. VIP scores >1 are significant and indicate important *X* variables that predict *Y* responses (lactational stages). **(F)** Permutation test of the PLS-DA model, indicating the absence of over-fitting in the model. The tolerance ellipse in scores scatter plots were drawn based on Hotelling's *T*^2^ with 95% confidence interval. PCA, principal component analysis; PLS-DA, partial least squares—discriminant analysis; Carbo, carbohydrate; PL, plasmin; SFA, saturated fatty acids; MUFA, monounsaturated fatty acids; LCPUFAs, long-chain poly-unsaturated fatty acids.

The relationship between fatty acids, macronutrients, and plasmin activities of milk were analyzed by a PCA model. The final model was summarized in seven PCs and described 78.6% of the total variance in the data. As shown in Figure 4A, the first PC and the second PC accounted for 29.0 and 12.6% of the total variation, respectively. Apart from one sample at the top left of the score plot, samples from the first week of lactation were clustered at the center and right side of the first PC, whereas samples from week 4, 8, and 24 are randomly distributed throughout the plot. According to the loading plot (Figure 4B), the first PC was positively driven by energy and fat, while it was negatively driven by trans C18:1 n-9, sum of SFA, and levels of C8:0, C21:0, C20:3 n-6, C20:0, C17:1, C17, C15:0, C14:1. The second PC showed a strong positive correlation with C20:3 n-3, C18:2 n-6, C22:2 and the sum of n-6 LCPUFAs. It can also be seen that fat and energy were highly correlated. Some fatty acids, for example, C20:1 and C18:1 n-9, C15:1 and *trans* C18:1 n-9, also showed high correlation with each other. By combining the score and loading plot, levels of protein, C18:0, and C16:1 as well as plasmin activities were correlated with samples from the first week of lactation that clustered at the center.

According to the PCA analysis, one extreme outlier (a week 1 sample) was excluded for supervised PLS-DA, for a better discrimination of milk samples between four lactational stages (Week 1, 4, 8, and 24). The model had two latent variables (*R*^2^*Y* = 0.35, *Q*^2^ = 0.16, *p* < 0.001 by CV-ANOVA) and the four groups are visualized in Figure 4C. It is clear that Week 1 samples (right side of *y*-axis) and Week 24 samples (left side of *y*-axis) can be discriminated from each other, whereas week 4 and week 8 samples can only be weakly discriminated from each other. The corresponding loading plot (Figure 4D) summarizes how the nutrients (*X*-variable) relate to each other as well as to lactational stage (*Y*-variable symbolized by a group dot). According to the location of *X*- and *Y*-variables, milk at one-week *postpartum* is characterized by high levels of protein and high plasmin activity, but low levels of C18:3 n-3 and C6:0. Conversely, milk at 24-weeks *postpartum* had low levels of protein, plasmin activities, and C20:4, while high levels of C18:3 n-3 and C6:0. Figure 4E with VIP values illustrates the important *X* variables (VIP >1) that predict *Y* responses (lactational stages), including protein, plasmin activity, C24:0, C18:3 n-3, and C22:2, followed by C20:4, C6:0, C20:3 n-3, C10:0, C22:0, C18:3 n-6, C18:2 n-6, Σ n-6 PUFAs, and C20:2.

A permutation test was used to evaluate the possible overfitting of the PLS-DA model, and a properly fitted model was identified as having values of *R*^2^-intercept <0.4 and a *Q*^2^-intercept <0.05. According to the permutation tests, the *R*^2^-intercept was 0.0987 and *Q*^2^-intercept was −0.208 (Figure 4F), indicating that the PLS-DA model had no overfitting and was credible. However, it is still notable that *Q*^2^ was below 0.5, indicating that the model showed weak predictability.

## Discussion

Levels of proteins, fat, carbohydrate, and energy measured in the study were in accordance with many previous reports, being in the ranges 0.9–1.9 g dl^−1^, 3.2–4.1 g dl^−1^, 6.2–7.8 g dl^−1^, and 65–70 kcal dl^−1^, respectively (5, 19, 43–46). Analysis of mid- to hind- milk collected in the study indicated that higher levels of fat and energy are present compared to that in foremilk (13). Our results support reports that levels of protein decrease during lactation and that levels of total carbohydrate are relatively static during lactation (5). The levels of selected human milk oligosaccharides (HMOs) in the same cohort of samples were measured by Overgaard Poulsen et al. (47); those results showed a higher level of HMOs but a lower level of lactose in the early stage of lactation compared to the later stages of lactation. The increased fat and energy content from 1 to 4 weeks after birth has also been reported by previous studies (19, 48). There was no significant impact of infant gender on fat and energy content in term milk, in agreement with previous reports (17, 23, 25). When considering BMI, our results showed that milk expressed by high BMI mothers contained higher levels of protein than that of lower BMI mothers at 4 weeks *post-partum*, which was not found in previous reports (29, 33).

The changes in plasmin activities during lactation was measured in the study as a supplemental information for protein levels, as it may be a potential factor influencing the protein quality and digestion of infants (49, 50). Trypsin and thrombin, which have been detected in human milk, have similar specificities to plasmin (51). However, thrombin does not interfere with the assay applied in our study (34) and levels of trypsin have been reported to be low in human milk (52). The plasmin activities in term milk were in the range 0.018–0.052 nmol AMC units min^−1^ ml^−1^, and the decreasing trend of plasmin activities and high correlation to milk protein levels during lactation are in accordance with previous reports (5, 6).

Concerning FAs in milk, the major SFA presented in human milk was palmitic acid and the major MUFA was oleic acid, and the abundance of these were in accordance with previous reports (38, 53, 54). AA (C20:4 n-6), EPA (C20:5 n-3), and DHA (C22:6 n-3) are widely reported LC PUFAs in human milk, because they are structural components of cellular membranes, and are incorporated in relatively large amounts during early growth of the brain and the retina (8). In our study, the levels of EPA (C20:5 n-3) and DHA (C22:6 n-3) were in the same range, whereas AA (C20:4 n-6) were lower compared to the literature, with reports of 0.04%−0.15% of total FAs for EPA (C20:5 n-3), 0.2%−0.7% of total FAs for DHA (C22:6 n-3), and 0.45%−0.8% of total FAs for AA (C20:4 n-6) (32, 35, 39, 53). The European Food Safety Authority (EFSA) recommends a voluntary addition of AA, DHA, and EPA in infant formula, with AA <1% of total FAs, DHA < total n-6 LCPUFAs (<2% of total FAs), and EPA < DHA (55), which is consistant with our results.

Linoleic acid (C18:2 n-6) is the precursor of AA (C20:4 n-6), while ALA (C18:3 n-3) is the precursor of EPA (C20:5 n-3) and DHA (C22:6 n-3) (8). As for AA, EPA, and DHA, there is a particular need for linoleic acid and ALA in the first 6 months of life in terms of the development of nervous system. EFSA requires that the ratio of linoleic acids to AA should between 5 to 15 (55), which is in accordance with our results that the ratio decreased during the first 6 month of lactation. The levels of linoleic acid (C18:2 n-6) were lower than previous reports (17%−18% total FAs), while ALA (C18:3 n-3) levels were comparable to a Croatian study and a Spanish study (0.7%−1.4%) (53, 56). The lower content of linoleic acid may be a result of the low maternal dietary intake of this FA (57). Mothers involved in this study had a higher intake of animal oil (low linoleic acids levels), but a lower intake of vegetable oil (high linoleic acids levels) (Supplementary Table S1). Differences in FA profiles results between studies may also result from analytical methods and from population characteristics (58, 59). The GC-FID method used in our study is sensitive with a low limit of detection. However, only 37 standards applied limited the FAs that can be identified.

Concerning the lactational effect on FA profiles, significant increases in levels of caproic acid (C6:0) and ALA (C18:3 n-3) agreed with the increase of caproic acid from colostrum to mature milk reported in a meta-analysis by Floris et al. (38). Decreased levels of AA (C20:4 n-6), DHA (C22:6 n-3), lignoceric acid (C24:0), erucic acid (C22:1 n-9), and docasadienoic acid (C22:2) during lactation found in our study have also been reported previously (38–40).

Statistically significant associations between maternal BMI and FAs in human milk were found in this study. Levels of some SFA, MUFA, and PUFA were higher, while levels of lignoceric acid (C24:0), AA (C20:4 n-6) and EPA (C20:5 n-3) were lower, in milk from mothers with BMI ≥25 kg m^−2^ at specific lactational stages. de la Garza Puentes et al. (41) reported higher pentadecanoic acid (C15:0) and heptadecanoic acid (C17:0) levels in milk from mothers with high BMI, as shown here. They also reported higher levels of caprylic acid (C8:0) from obese mothers (BMI ≥ 30 kg m^−2^) compared to mothers with BMI between 25 and 30 kg m^−2^ in human milk. However, a statistically significant difference (*p* < 0.05) was only found in colostrum in their study, not in transitional or mature human milk. In terms of LC PUFA, higher levels of pentadecanoic acid (C15:0), heneicosanoic acid (C21:0) and trans-linolelaidic acid (trans C18:2 n-6) were found in mothers with higher BMI than mothers with lower BMI, similar to our results; however, they did not find statistical significance (*p* > 0.05) (60). Instead, these authors found significantly higher palmitic acid (C16:0) and significantly lower oleic acid (C18:1 n-9) levels in milk of mothers with BMI ≥ 25 kg m^−2^, which was not observed in our study. A recent study showed that milk from these two BMI groups could be distinguished by fatty acid profiles, and C21:0 and C24:0 had high discrimination coefficients, which is similar to our study (42).

To our knowledge, few reports have related levels of FAs to infant gender. Significant relationships were found between infants' gender and some FAs in this study. The major impact of infant gender was observed after 8- or 24-weeks *post-partum* in milk. Higher levels (mg/g fat) of ALA (C18:3 n-3) and ∑ n-3 LCPUFAs at week 8 *post-partum*, some SFAs and some MUFAs at week 24 *post-partum* were found in milk for female infants compared to milk for male infants (Supplementary Figure S4). Thakkar et al. (61) also reported higher myristic acid (C14:0), ALA (C18:3 n-3), and total SFA levels in milk for female offspring than for male offspring during a 120-day lactational observation; however, the difference was not significant (*p* > 0.05). They also reported significantly higher levels of linoleic acid (C18:2 n-6), 20:2 n-6 (eicosadienoic acid), and total PUFA in milk for female infants than milk for male infants, which was not observed in our study. One recent study did not find significant relationships between infant gender and FA profiles (62). Interestingly, in terms of the sex-bias of nervonic acid (C24:1) levels found in our study, this FA has been reported to decrease during lactation (not found in this study) with higher abundance in preterm human milk (40, 63). Nervonic acid is important for white matter brain development and thus contributes to the central nervous system of infants (63), and has been reported to be present at significantly higher levels in human milk compared to infant formula (64). The significance of variations in levels of this FA may need further investigation.

Multivariate statistical analysis provided a whole picture for the nutrients rather than only individual profiles (Figure 4). Although PCA could only weakly discriminate milk samples from four lactational stages using the first two PCs, it was clear that some fatty acids are closely located and thus highly correlated and that total fat content is negatively correlated with some SFA (C15:0, C17:0, C20:0, and C21:0). Interestingly, caproic acid (C6:0), a medium-chain fatty acid that increased in level during the first 6 months of lactation, showed an opposite projection on PC1 compared to other fatty acids in the PCA loading plot.

Supervised PLS-DA improved the discrimination of milk samples from lactational groups, and clearly separated week 1 and week 24 samples. The *Q*^2^ of the model is low, which could be a result of the poor discrimination of samples at lactational week 4 and week 8. Except for a few fatty acids (such as C24:0, C18:3 n-3), fatty acids showed lower contribution to predict milk from different lactational stages compared to protein and plasmin activities. This result indicates that milk proteins and proteases, moreso than fatty acids, are highly impacted by lactation stage.

The strength of this study is that it provides an overall profile of milk composition and plasmin activity and detailed comparison of FAs, as affected by lactational time points, gender, and BMI. Limitations of our study that could be addressed in future work is that numbers of mothers in different cohort are more closely balanced, information on diet could be more specific, and only fatty acids provided in the form of standards were analyzed.

## Conclusion

There was a significant influence (*p* < 0.05) of lactation stage on levels of protein and plasmin activity in human milk during lactation. Pre-pregnancy BMI influenced the protein content of human milk. In addition, there was significant influence (*p* < 0.05) of lactation, pre-pregnancy maternal BMI and infant gender on individual FA profile in term human milk. *Cis*-C20:1 and C23:0 levels decreased during the first 4 weeks after birth, while those of C18:3 n-6 increased during the first 4 weeks but decreased from 4 to 24 weeks after birth. Levels of 12 fatty acids were higher in milk from mothers with high BMI at specific lactational stages, while only levels of C22:2 were lower in milk from mothers with high BMI from 4 to 8 weeks after birth. This study updates knowledge on the impact of human milk lactation, pre-pregnancy maternal BMI, and infants' gender on human milk macronutrients, fatty acid profiles, and protease activities, providing an overall picture on human milk macronutrients that can be useful in improving infant nutrition, for example, through strategies for targeted fortification of human breast milk in infancy.

## Data availability statement

The raw data supporting the conclusions of this article will be made available by the authors, without undue reservation.

## Ethics statement

The studies involving human participants were reviewed and approved by Clinical Research Ethics Committee of the Cork Teaching Hospitals. The patients/participants provided their written informed consent to participate in this study.

## Author contributions

Conceptualization: AR, AK, and CS. Methodology: FM, TU-L, and EL. Formal analysis: FM, TU-L, and TD. Resources: GM and C-AO'S. Data curation and writing—original draft preparation: FM. Writing—review and editing: TU-L, FM, and AK. Supervision: AK. Funding acquisition: AK and CS. All authors have read and agreed to the published version of the manuscript.

## Supplementary material

Supplementary Table S1 Diet information of mothers within 24 h of each expression.

Supplementary Figure S1 Macronutrient and energy levels in term human milk from mothers with normal weight (BMI < 25 kg m^−2^) and from overweight mothers (BMI ≥ 25 kg m^−2^) during the first 6 months of lactation. Lines extending vertically from the boxes indicate variability outside the upper and lower quartiles. Mild outliers (°, 1.5–3 times outside the interquartile range), extreme outliers (×, 3 times outside the interquartile range), and statistical significance (*p* < 0.05) between BMI groups are indicated. Normal, normal weight; OW, overweight.

Supplementary Figure S2 Macronutrient and energy levels in term human milk from mothers who gave birth to female infants and mothers who gave birth to male infants during the first 6 months of lactation. Lines extending vertically from the boxes indicate variability outside the upper and lower quartiles. Mild outliers (°, 1.5–3 times outside the interquartile range), extreme outliers (×, 3 times outside the interquartile range), and statistical significance (*p* < 0.05) between gender are indicated.

Supplementary Figure S3 Fatty acids levels in term human milk from mothers with normal weight (BMI < 25 kg m^−2^; white bar) and those with BMI ≥ 25 kg m^−2^ (gray bar) during the first 6 months of lactation. Lines extending vertically from the boxes indicate variability outside the upper and lower quartiles. Mild outliers (°, 1.5–3 times outside the interquartile range) and statistical significance (*p* < 0.05) between BMI groups are indicated.

Supplementary Figure S4 Fatty acids levels from mothers who gave birth to female infants (white bar) and mothers who gave birth to male infants (gray bar) during the first 6 month of lactation. Lines extending vertically from the boxes indicate variability outside the upper and lower quartiles. Mild outliers (°, 1.5–3 times outside the interquartile range) and statistical significance (*p* < 0.05) between gender are indicated.
