# Supplementary material for: A longitudinal study of fatty acid profiles, macronutrient levels, and plasmin activity in human milk
Source: Front Nutr. 2023 May 9;10:1172613. doi: 10.3389/fnut.2023.1172613 (PMC10203173; doi:10.3389/fnut.2023.1172613)

Supplementary Material

# Supplementary Tables

**Table S1** Diet information of mothers within 24 hours of each expression.

|  | Diet information of term infant mothers | | | |
| --- | --- | --- | --- | --- |
| Lactational stage (week) | 1 | 4 | 8 | 24 |
| N | 74 | 74 | 64 | 45 |
| Pump/Manual (%Pump) | 46 | 59 | 66 | 60 |
| Exclusive breast-feeding （% Exclusive) | 82 | 74 | 73 | 56 |
| Volume of formula per day if supply (mL) | 15-750 | 30 - 500 | 100-960 | 30-600 |
| Oily fish (% Yes) | 11 | 15 | 8 | 13 |
| White fish (% Yes) | 0 | 9 | 5 | 7 |
| Red meat (% Yes) | 66 | 49 | 56 | 47 |
| Poultry (% Yes) | 55 | 57 | 66 | 69 |
| Vegetable oil (% Yes) | 42 | 35 | 23 | 31 |
| Olive Oil (% Yes) | 46 | 46 | 48 | 49 |
| Butter (% Yes) | 80 | 91 | 80 | 67 |
| Seeds (% Yes) | 39 | 50 | 44 | 53 |
| Nuts (% Yes) | 35 | 39 | 42 | 29 |
| Vegetables (% Yes) | 96 | 93 | 94 | 91 |
| Dairy Products (% Yes) | 91 | 93 | 84 | 87 |
| Bread (% Yes) | 95 | 97 | 91 | 76 |
| Beans, pulses (% Yes) | 19 | 18 | 14 | 16 |
| Supplements (% Yes) | 55 | 57 | 59 | 39 |
| Alcohol (% Yes) | 5 | 4 | 6 | 5 |

Fig. S1
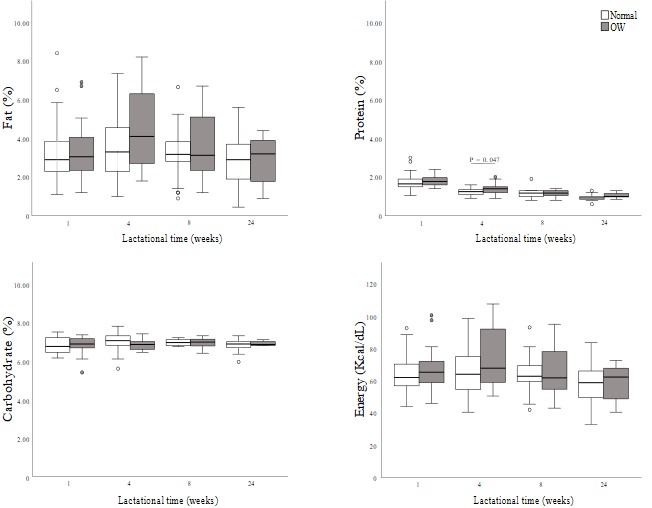


Fig. S2


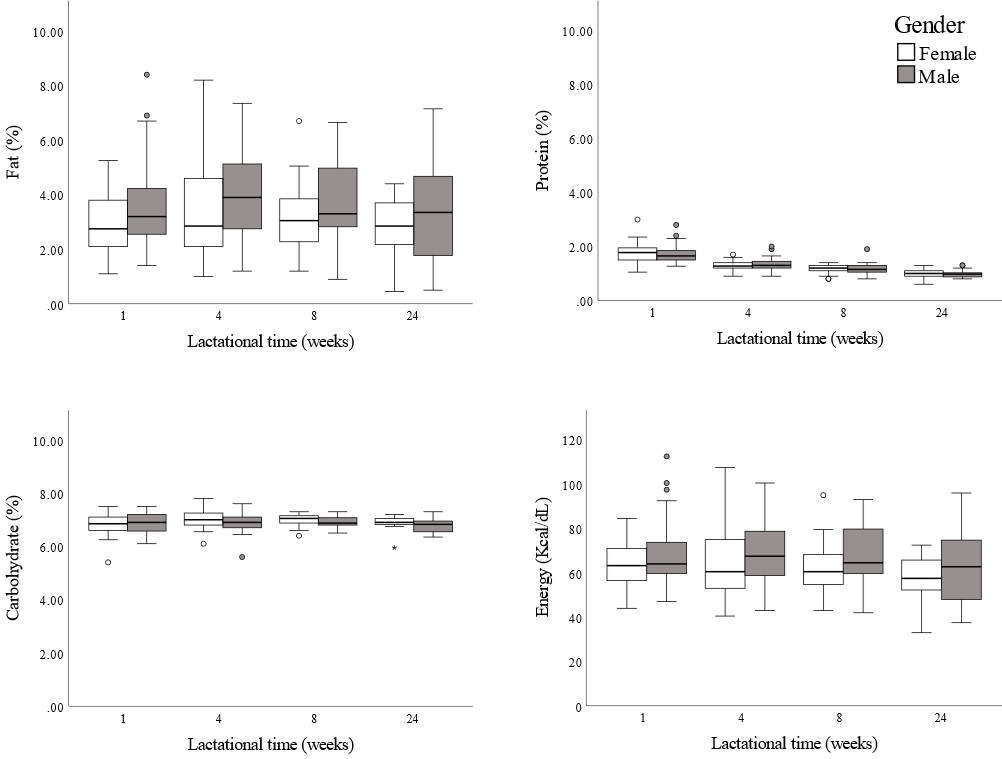


Fig.S3


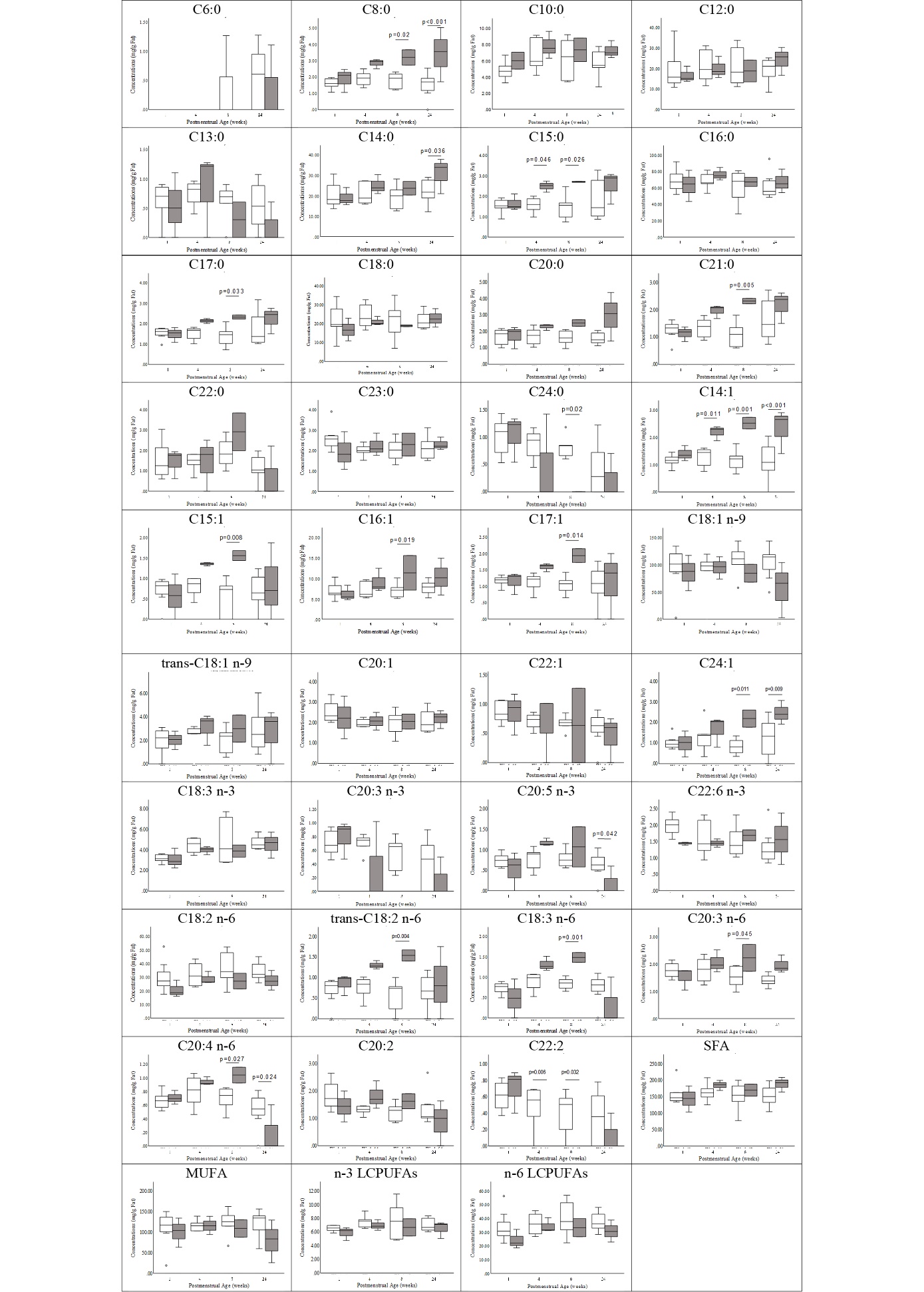


Fig. S4


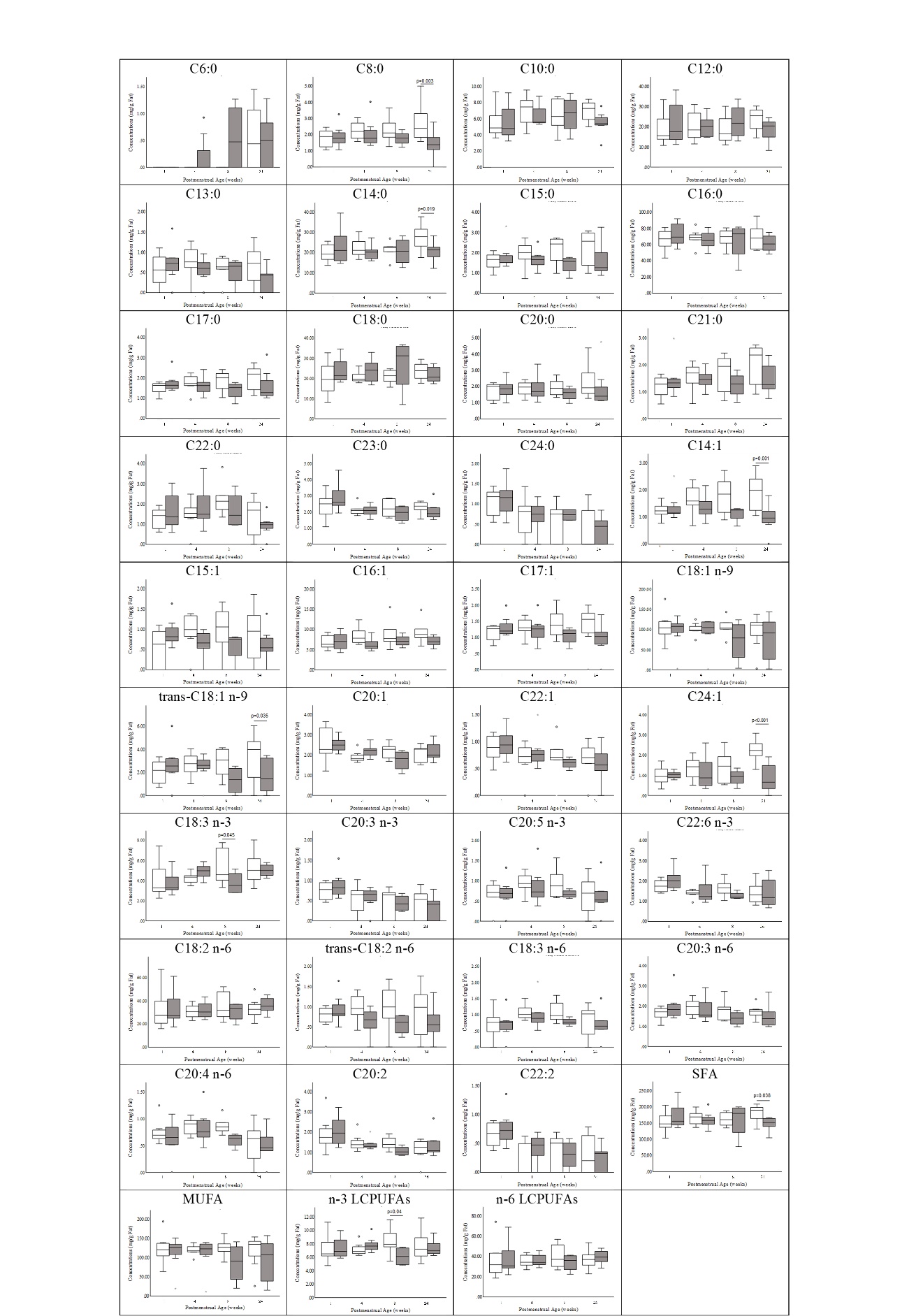

Supplement: Supplementary Table S1 — Diet information of mothers within 24 h of each expression. [file Data_Sheet_1.docx]
